# Editorial: Microbial Safety in Water Resources

**DOI:** 10.3389/fmicb.2018.03064

**Published:** 2018-12-10

**Authors:** Pei-Ying Hong, Timothy R. Julian, Muhammad Raihan Jumat

**Affiliations:** ^1^Water Desalination and Reuse Center, Biological and Environmental Science & Engineering Division, King Abdullah University of Science and Technology, Thuwal, Saudi Arabia; ^2^Eawag, Swiss Federal Institute of Aquatic Science and Technology, Dübendorf, Switzerland; ^3^Department of Epidemiology and Public Health, Swiss Tropical and Public Health Institute, Basel, Switzerland; ^4^University of Basel, Basel, Switzerland

**Keywords:** wastewater treatment, water reuse, water quality, molecular methods, microbial contaminants

The scientific community can help to advance wastewater reuse in two important ways: First, through the exploration of new treatments and technologies that allow use of safe water supply alternatives, and second, through development and use of new methods that improve insights on water quality. Examples of these two contributions are provided here in the Frontiers Research Topic *Microbial Safety in Water Resources*.

Wastewater treatment technologies serve as important engineering barriers to remove majority of the contaminants from wastewater, hence achieving safe water reuse or disposal to the natural environment. The conventional wastewater treatment process include clarifiers or sedimentation tanks, activated sludge processes, and disinfection. In recent years, membranes (e.g., microfiltration) are also retrofitted into activated sludge tanks to form aerobic membrane bioreactors, which in turn improve solid-liquid separation and hence achieve high effluent quality.

In most instances, the above described wastewater treatment processes are operated as centralized facilities. However, decentralized facilities are gaining in favor due to decreased capital costs, reduced reliance on sewage infrastructure, and potential for resource recovery at a local-scale. Depending on the treatment process, a well-operated decentralized process can perform as well as that of centralized treatment. To exemplify, Nguyen et al. evaluated on-site treatment of wastewater using granular activated carbon, chlorination and electrolysis, demonstrating 5-log inactivation of *Escherichia coli*. The study also demonstrates how modular technologies should be explored to tackle emerging contaminants present in untreated wastewaters. If a wastewater treatment process is designed with modularity in mind, the process can adapt dynamically to meet the current needs. This concept of modularity is also reviewed by Barancheshme and Munir in a discussion of treatment options to combat antimicrobial resistance threats arising from wastewaters.

Clearly, a well-operated treatment plant remains an important barrier to reduce contaminant dissemination from wastewaters into the environment. However, recent studies showed that some bacterial strains developed strategies to survive treatment and environmental stressors (Al-Jassim et al., 2017; Mantilla-Calderon and Hong, 2017; Jumat et al., 2018). Trigui et al. discussed this concept by examining differences in viability rates between two *Campylobacter jejuni* strains. The *C. jejuni* strain isolated from oligotrophic water was able to survive better in a freshwater medium than the other strain, potentially due to the observed higher resistance to oxidative stress and bile salts. Interestingly, genes involved in resisting against oxidative stress and bile salts were induced, hence conferring a protective effect.

The results from Trigui et al. and many other studies demonstrate that understanding the microbial ecosystems and the microbial behaviors will improve effective mitigation measures downstream of wastewater treatment. Such measures include storing treated wastewaters in an evaporation pond and exposing the waters to natural sunlight. Sunlight achieves antimicrobial effect via both direct DNA damage and radical oxidative species (ROS)-mediated damage. Depending on the depth of the pond, solar exposure can occur either under oxic or anoxic conditions, resulting in differences in decay rates and gene expression for the same bacterium. This concept is explored by McClary and Boehm, who used *Staphylococcus aureus* to demonstrate a different response toward oxygen-dependent and oxygen-independent photostress.

Treatment plants can also utilize the more conventional chlorine or other disinfectants (e.g., chlorine dioxide, UV radiation, and heat lysis) to further inactivate remnant microbial contaminants. Different disinfection strategies inactivate microbial contaminants via different mechanisms. Zhong et al. explained that chlorine and chlorine dioxide inactivate Echovirus 11 by inhibiting the binding interactions between viruses and host cells. Echovirus strains that are resistant to chlorine exhibit cross resistance to chlorine dioxide but were susceptible to UV, sunlight and heat treatment due to the differences in disinfection strategy. Their findings suggest a need to complement different disinfection strategies for improved viral removal.

The scientific community can also help advance wastewater reuse through the development and use of novel methods for water quality monitoring. Novel methods are needed to gain insight into the presence, quantity, and dynamics of new and emerging pollutants, better characterize microbial populations including pathogen ecology, and improve specificity and sensitivity of existing, primarily culture-based, tools. For example, water quality monitoring often requires monitoring for culturable bacteria, such as *E. coli*, fecal coliforms, total coliforms, and/or heterotrophs. Culture-based methods suffer numerous limitations, including limited or no correlation with the presence of pathogens, and susceptibility to false-positives. Recent work has highlighted that US-EPA approved media for detection of fecal coliforms and *E. coli* can be particularly prone to false-positives (Olstadt et al., 2007; Zhang et al., 2015). This can generate unnecessary alarm and operational costs for water utilities.

Four of the papers published in this Research Topic demonstrate and/or implement new water quality monitoring technologies to gain new understanding on water quality. Specifically, online flow cytometry, 16S rRNA gene-based amplicon sequencing, genome characterization, and metagenomics. Besmer et al. demonstrated the use of online flow cytometry with an optimized monitoring strategy to detect precipitation-induced microbial peak loads in karstic spring waters. This method could potentially be useful when applied to climate-change induced precipitation that may differ from predictable rainfall patterns on a local scale. Similarly, Uprety et al. utilized 16S rRNA gene-based amplicon sequencing to monitor microbial dynamics in groundwater before and after an earthquake. By looking into the relative abundances of microbial groups, the authors could determine a short-term perturbation on the indigenous groups that eventually restore with time after implementation of sanitation practices.

In recent years, decreasing costs in next generation sequencing have resulted in an increase in the use of metagenomics to elucidate water quality. Ng et al. utilized metagenomics to elucidate the diversities and average relative abundance of antibiotic resistance genes present in hospitals and untreated municipal wastewaters. The authors further assembled scaffolds from the raw sequencing reads, and identified that most of the ARGs are associated with mobile genetic elements that can aid in horizontal transfer of resistance genes among bacterial populations.

This technique of assembling scaffolds was further exemplified by Zhang et al. By first performing very deep metagenomics sequencing on drinking water samples, sufficient coverage was achieved to assemble raw reads into complete draft genomes of 9 bacterium. Further annotation of the draft genomes revealed pathogenic characteristics and for some, CRISPR-Cas genetic signatures, present in the drinking water samples. Such insights would not have been discovered by conventional culture techniques, hence reiterating the usefulness of exploring new molecular methods.

In summary, articles in this Research Topic exemplify how the scientific community can work toward addressing water scarcity by a two-pronged approach—first, to explore alternative water supplies and ensuring that these new waters are safe for use; second, utilizing new methods to provide comprehensive insights on water quality, which would in turn advance water reuse and management programs in a safe and sustainable manner.

## Author Contributions

P-YH conceived the outline, wrote and edited the manuscript. TJ and MJ contributed to the writing and editing of the manuscript. All authors contributed to the overall framing, writing, and revision of this manuscript.

### Conflict of Interest Statement

The authors declare that the research was conducted in the absence of any commercial or financial relationships that could be construed as a potential conflict of interest.
